# Regional cerebral blood flow as predictor of response to occipital nerve block in cluster headache

**DOI:** 10.1186/s10194-021-01304-9

**Published:** 2021-08-12

**Authors:** Sonia Medina, Norazah Abu Bakar, Owen O’Daly, Sarah Miller, Elena Makovac, Tara Renton, Steve C. R. Williams, Manjit Matharu, Matthew A. Howard

**Affiliations:** 1 Department of Neuroimaging, King’s College London, Institute of Psychiatry, Psychology and Neuroscience, Box 89, De Crespigny Park, London, SE5 8AF UK; 2 Wolfson Centre for Age-Related Diseases, King’s College London, London, UK; 3 Department of Oral Surgery, King’s College London, London, UK; 4 Headache and Facial Pain Group, UCL Queen Square Institute of Neurology, Queen Square, London, UK

**Keywords:** Cluster headache, Greater occipital nerve block, Regional cerebral blood flow, Arterial spin Labelling, Trigeminal cephalgia

## Abstract

**Background:**

Cluster headache is an excruciating disorder with no cure. Greater occipital nerve blockades can transiently suppress attacks in approximately 50% of patients, however, its mechanism of action remains uncertain, and there are no reliable predictors of treatment response. To address this, we investigated the effect of occipital nerve blockade on regional cerebral blood flow (rCBF), an index of brain activity, and differences between treatment responders and non-responders. Finally, we compared baseline perfusion maps from patients to a matched group of healthy controls.

**Methods:**

21 male, treatment-naive patients were recruited while in a cluster headache bout. During a pain-free phase between headaches, patients underwent pseudo-continuous arterial spin labelled MRI assessments to provide quantitative indices of rCBF. MRIs were performed prior to and 7-to-21 days following treatment. Patients also recorded the frequency of their headache attacks in a daily paper diary. Neuropsychological assessment including anxiety, depression and quality of life measures was performed in a first, scanning free session for each patient.

**Results:**

Following treatment, patients demonstrated relative rCBF reductions in posterior temporal gyrus, cerebellum and caudate, and rCBF increases in occipital cortex. Responders demonstrated relative rCBF increases, compared to non-responders, in medial prefrontal cortex and lateral occipital cortex at baseline, but relative reductions in cingulate and middle temporal cortices. rCBF was increased in patients compared to healthy controls in cerebellum and hippocampus, but reduced in orbitofrontal cortex, insula and middle temporal gyrus.

**Conclusions:**

We provide new mechanistic insights regarding the aetiology of cluster headache, the mechanisms of action of occipital nerve blockades and potential predictors of treatment response. Future investigation should determine whether observed effects are reproducible and extend to other headache disorders.

## Introduction

Cluster headache (CH), a member of the group of trigeminal autonomic cephalgias, is an excruciating condition. It is characterised by strictly unilateral orbital, supraorbital or temporal headaches that severely compromise the quality of life of those who suffer from it. While there are a number of treatments available to alleviate CH symptoms, at least partially [1], further development is still needed to achieve complete suppression of headache attacks and effective management of commonly associated psychological symptoms (e.g. anxiety, depression).

It is still unclear how some of these therapies work in CH treatment responders, which suggests the involvement of several interrelated neural processes which require better characterisation. Greater occipital nerve blockade (GONB) is a relatively successful therapy for suppressing CH attacks with minimal side effects [2]. GONB action is theorised to reduce afferent signalling from the occipital nerve to the sensory trigeminal fibres at the level of the nucleus caudalis, however, the degree of such inhibition is not directly reflected in a proportional reduction of CH symptoms [3]. A well-defined model that explains how a GONB stops headache attacks, and why it is effective in only a portion of patients who receive it, is yet to be proposed.

Neuroimaging has significantly facilitated our understanding of putative brain mechanisms underpinning CH [4–7]. Functional magnetic resonance imaging (fMRI) and in particular blood-oxygen-level dependent (BOLD) fMRI, can describe differences in activity and connectivity between CH patients and healthy controls [8], both in the resting state and during headache attacks [9, 10], pointing towards the hypothalamus as a key area involved in triggering headache attacks during bouts, as well as in marking the beginning and end of bouts in episodic CH patients, causing the circadian nature of CH symptoms. Nevertheless, these findings continue to be debated, as it remains unclear whether results incorporate the hypothalamus and/or the neighbouring ventral tegmental area (VTA) as the areas responsible for those differences [11]. These contentions are compounded by the small size of these structures and the limited spatial resolution of fMRI. In fact, chronic pain largely relates to spontaneous, low frequency fluctuations, for which arterial spin labelling (ASL) is more optimally sensitive, as it can identify changes in low frequency brain activity via quantification of regional cerebral blood flow (rCBF) as a proxy of resting brain activity in relation to chronic pain [12]. Evidence of rCBF changes in CH patients after GONB should therefore provide important new mechanistic insights.

Previous studies have reported decreased metabolism [13] and grey matter volume (GMV) [14, 15] in the prefrontal cortex (PFC) in CH patients in comparison to healthy controls, as well as negative correlations between PFC GMV and disease duration [16]. GMV in medial PFC has been considered as predictor of response to treatments for depression [17] and anxiety disorders [18], both common comorbidities in CH. Accordingly, we hypothesised that prefrontal rCBF at baseline could relate to the capacity of treatment response, ultimately contributing to differential responses to GONB; therefore we anticipated that prefrontal CBF at baseline would differ between CH patients and healthy controls, as well as between those who respond positively to GONB (i.e. responders) and treatment non-responders.

Here, we explored i) rCBF changes in CH patients following their first GONB treatment to further understand the mechanisms of action of GONB, ii) differences in rCBF across CH patients at baseline during interictal phase in relation to response to GONB treatment, and iii) brain perfusion differences between CH patients at baseline and healthy controls. We hypothesised that a) GONB would result in rCBF differences throughout the brain, b) patterns of baseline rCBF would be useful predictors of treatment response, particularly in the PFC, and c) rCBF would differ between patient and control groups.

## Materials and methods

### Eligibility, groups and screening

Twenty-one CH patients (age range: 20–55 years, mean = 37.5 ± SD = 8.9) were recruited at The National Hospital for Neurology and Neurosurgery in London. Inclusion criteria were: (i) being a male participant; females were excluded from the study to avoid confounds relating to fluctuations in female hormonal levels within and between sessions [19]; (ii) patients diagnosed with CH according to diagnostic criteria in effect at the time of the study [20] and receiving their first GONB as part of their medical plan; (iii) age range 18–65 years; (iv) in case of being on preventive medication treatment, a stable dose for a minimum of 1 month [21]; no history or evidence of psychosis, psychological disease, use of recreational drugs or excessive caffeine consumption (i.e. more than six cups of caffeinated drinks per day) [21]; no existing major medical problems aside from CH (e.g. heart disease) and (vii) normal criteria for MRI scanning. Having an abortive treatment within the last 12 h prior to the scanning sessions was also an exclusion criterion, apart from oxygen treatment. Although existing evidence suggests that brain perfusion likely returns to baseline only a few minutes after a state of hyperoxia, especially under higher concentrations of oxygen [22, 23], oxygen treatment was allowed up to 1 h before each scanning session to avoid any confounding effects.

In addition, data from seven male, age-matched, physically and psychologically healthy controls from previous studies were included in the last data analysis set. All healthy controls had provided prior written consent for their MRI data to be used in later studies. Exclusion criteria for the recruitment of healthy volunteers included history of brain injuries, hypertension, any psychiatric or neurologic disease, alcohol or drug abuse, insomnia, obstructive sleep apnea, narcolepsy, or restless legs syndrome. Any volunteers that were acutely ill, with fever and malaise, were excluded or rescheduled for examination following complete recovery.

### Study design

**Fig. 1:**
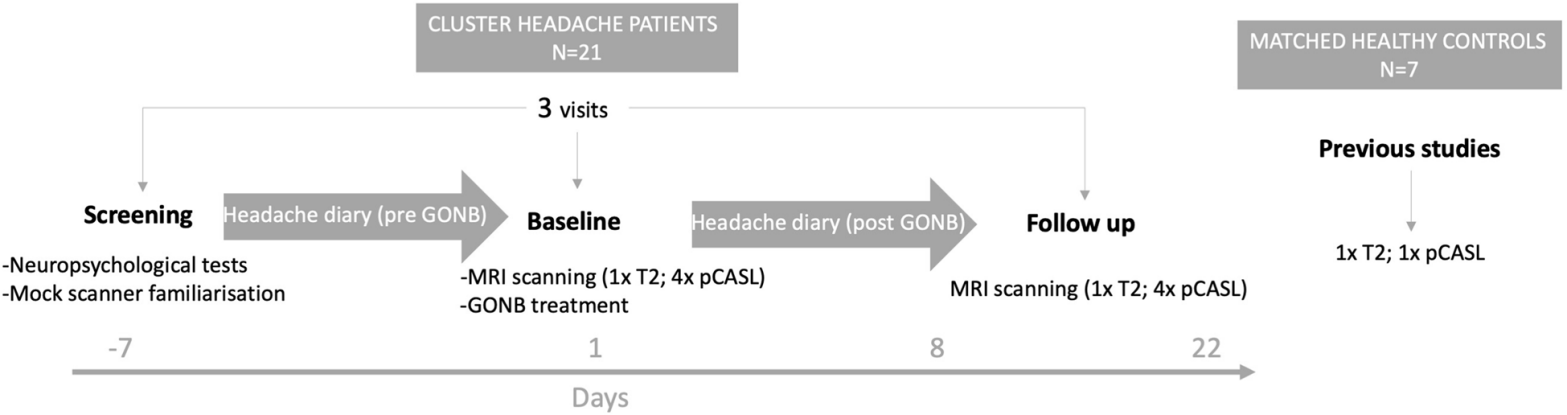
Experimental design. Twenty-one male patients attended three sessions, where the first session consisted of a clinical and neuropsychological screening and mock scanner familiarisation; during the second session baseline MRI measurements were collected and following this, GONB treatment was administered; the third sessions served as a follow up visit, where MRI measurements were collected once again. In addition, PCASL and structural data were acquired from a group of seven matched healthy controls, using identical pulse sequence in the same scanner, to permit further comparisons. All three visits were completed within a time window of approximately 30 days

A prospective, open-label study was carried out. For all patients, the study required three visits to the imaging centre; (i) a neuropsychological screening and a mock scanning session to familiarise patients to the scanner environment; (ii) a baseline MRI scanning session (including structural T2-weighted images and pCASL measurements) followed by GONB treatment; (iii) a third session, taking place between 7 and 21 days following treatment to examine treatment effects once the effects of the injection were allowed to emerge. At this final session, an MRI scanning session identical to the first was performed (Fig. 1). In addition, for the entire duration of the study, patients were requested to record, on a daily basis, the number of CH attacks experienced during that day, as well as their duration and severity. All visits were scheduled during patients’ bouts in the case of episodic CH (ECH) patients and between CH attacks (i.e. pain-free). The average duration of ECH bouts was taken into account to make sure the last session did not occur during a natural remission of CH attacks towards the end of a bout, as this could confound the results regarding GONB response. Patients who experience a definite response to GONB after a week were called in earlier to complete their follow up session; on the contrary, patients showing partial/no response to GONB a week after treatment were called in later to ensure enough time was provided for the effects of GONB to be seen and they were not wrongly labelled as non-responders. During MRI scanning, patients and healthy controls were instructed to remain awake in order to control for their level of alertness.

### Study procedures

Neuropsychological assessments were carried out at The National Hospital for Neurology and Neurosurgery in London. All the scanning sessions and GONB were performed at the Centre for Neuroimaging Sciences, Institute of Psychiatry, Psychology and Neuroscience, King’s College London.

### Neuropsychological assessment

All CH patients underwent a neuropsychological assessment on the first visit, including the Hospital Anxiety and Depression Scale (HADS) [24] and MOS 36-Item Short-Form Health Survey (SF-36) to assess patients’ quality of life [25].

### GONB treatment

The greater occipital nerve block procedure comprised 80 mg methylprednisolone and 2 ml of 2% lidocaine, injected in the suboccipital area at a point lying on the medial third of a line drawn between the inion and mastoid process ipsilateral to the pain.

### MRI acquisition and pre-processing

MRI imaging was acquired on a 3 T General Electric Signa HDX whole-body MRI scanner, equipped with an 8-channel, receive-only, phased-array head coil. All patients and healthy controls had an axial T_2_-weighted 3D fast-recovered fast spin-echo (FSE) pulse sequence with slice thickness = 2 mm, repetition time = 4380 ms, echo time = 55.44 ms, flip angle = 90°, field of view = 240 mm^2^, and matrix size = 320 × 320. For brain perfusion measurement, a pCASL sequence was used (8 shots, 512 points per spiral arm, repetition time = 1635 ms, echo time = 5.222 ms, pulse duration = 500 μs, pulse gap = 1500 μs, post-labelling delay = 1.5 s, voxel size = 1.875 × 1.875x3mm^3^). For all CH patients, regional cerebral blood flow (rCBF) quantification was repeated four times each session. Further details on rCBF computation can be found at https://www.kcl.ac.uk/ioppn/depts/neuroimaging/research/pain/pCASLdetail.pdf. All MRI measurements were carried out during patients’ pain-free interictal phases.

Image pre-processing was performed using the FMRIB Software Library (FSL). The first step was skull stripping and segmentation of structural images using FSL BET and FIRST, respectively. T2 scans were normalised to a Montreal Neurological Institute (MNI) T2 template using linear and non-linear registration tools FLIRT and FNIRT respectively. We performed co-registration of pCASL images to native-space T2 skull-stripped scans; the resulting transformation matrix was then inverted and used to co-register normalised T2 scans to pCASL images. Stripped, co-registered pCASL scans were warped into MNI space using linear and non-linear transformation parameters derived resulted from the high-resolution T2-weighted structural images. Images were finally spatially smoothed with an 8 mm FWHM isotropic Gaussian kernel using Statistical Parametric Mapping software (SPM) version 12. Quality assurance was manually performed to identify artefacts (e.g. co-registration failures) at each pre-processing step.

### Statistical analyses

#### Behavioural data

The mean and standard deviation of the number of weekly CH attacks before and after GONB (according to their Headache Diary notes) were computed for each patient. Patients were then divided in two groups: the *responders* group included patients who experienced a 50% or greater reduction in the average of weekly CH attacks after GONB treatment; the remaining patients were included in the *non-responders* group. Results from HADS and SF-36 subscales were compared between the *responders* and *non-responders* group by means of an independent samples t-test.

#### Neuroimaging data

Group wise statistical analyses of pre-processed pCASL images were carried out in SPM 12 using a mass univariate general linear model approach. For inference, an initial uncorrected cluster-forming height threshold was set to *p* < 0.001. Results were Family-Wise Error (FWE) corrected on the basis of cluster extent at *p* < 0.05 according to Random Field Theory. An explicit grey matter mask template was included in all the designs. Strictly for exploratory purposes, whole brain changes across patients after treatment are also displayed at a less stringent uncorrected cluster-forming height threshold = 0.005. Effect size statistics (Cohen’s d) were computed as a function of the t value for each contrast and the sample size, accounting for both paired [26] or independent samples [27] model designs. In order to avoid inflated effect sizes, that may be biased due to small sample sizes (i.e.*n* < 20), Hedge’s g statistic for corrected effect size were also calculated as a function of Cohen’s d results [28].

#### Changes in rCBF after treatment across patients

In order to examine regional differences in CBF before and after treatment, we performed a repeated-measures analysis of variance (ANOVA) with three factors: Subjects, Treatment (pre/post treatment) and Scan (with four levels, one per CBF map). Despite the fact that all patients were scanned during an interictal phase in a pain-free state, CBF maps from episodic CH and chronic CH patients were compared at baseline via an independent sample t-test, to assess the appropriateness of their inclusion in subsequent modelling as a single sample. No significant differences were identified. As CH attacks are presented unilaterally, we investigated CBF maps from patients reporting attacks on their right side, compared to those reporting attacks on their left, using an independent sample t-test, in order to rule out confounding effects of the headache side. Since no significant differences were observed, in subsequent models information about the laterality of attacks was included in the design as a nuisance covariate. Patients age, duration of CH (measured in number of years from the first CH attack to the moment of first visit) and global CBF signal were also included as additional nuisance covariates. We examined the main effect of Treatment via *pre-treatment </> post-treatment* contrasts.

#### Responders vs non responders to GONB treatment

Scans acquired before treatment for the *responders* and *non-responders* group were compared via a two-way analysis of variance (ANOVA) with two factors: Treatment response (responders/non responders) and Scan (four levels, one per CBF map) to determine whether baseline rCBF could predict response or non-response to GONB treatment in CH. Global CBF, age, duration of CH, global white matter volume and global cerebrospinal fluid volume (CSF) (both measured in millilitres) were included in the analysis as additional nuisance covariates. Two analyses were performed with patients’ pre and post treatment images respectively.

In order to test our a priori hypothesis regarding prefrontal local CBF increases in the responders group compared to non-responders, we performed a small volume correction (SVC) for the contrast *‘responders > non-responders’*. The frontal cortex region of interest (ROI) was chosen from a predefined mask in SPM12.

#### CH patients vs healthy controls

Baseline pCASL data from all patients were compared with data from an available database of age matched healthy controls, to determine rCBF differences that relate to CH. In this case, only the first CBF map for each CH individual was used for analysis, due to data availability limitations from healthy controls. Global CBF, global white matter volume, global CSF volume, and number of months passed from the scanner acquisition date to the analysis date were added to the model as nuisance covariates.

## Results

Two patients failed to complete the last session, and one was excluded due to unrecoverable motion artefacts in MRI data. Data from a total of 18 patients were included in the data analysis.

### Behavioural data

#### Headache diary data

One patient did not provide information for the Headache Diary; results from a total of 17 patients were included in the behavioural data analysis (Tables 1 and 2). Overall, 52% of patients (*n* = 9) were considered *responders* to treatment, experiencing a reduction in weekly CH attacks greater than 50%, six of whom became completely pain free. Eight patients were designated as *non-responders*; within this group, two patients reported a reduction in weekly attacks lower than 50%, no changes were reported by two patients and remaining subjects reported an increased number of attacks after treatment. Response to GONB could be determined 1 week after GONB in most cases, except for one patient who showed a positive response in terms of reduction of average weekly attacks after 3 weeks.

**Table 1:** Demographic and main clinical data from CH patients included in behavioural data analyses (*N* = 17)

Patient	Age	Duration CH	CH side	Type of CH	Preventive medication	Acute medication
1	39	14	Right	Episodic	–	Triptan
						Oxygen
2	29	7	Right	Episodic	Verapamil	Triptan
3^a^	46	5	Right	Episodic	–	Triptan
						Oxygen
4	34	17	Left	Episodic		Triptan Oxygen
5	50	10	Right	Chronic	Verapamil	Triptan
					Topiramate	Oxygen
					Tricyclic antidepressants	
6	64	42	Right	Episodic	–	Triptan
						Oxygen
7	36	7	Right	Chronic	–	–
8	35	7	Right	Episodic	–	Triptan
						Oxygen
9	32	10	Left	Chronic	–	Triptan
						Oxygen
10	34	11	Right	Episodic	–	Oxygen
11	34	6	Right	Chronic	Verapamil	Triptan
						Oxygen
12	43	26	Right	Episodic	–	Triptan
						Oxygen
13	55	25	Right	Episodic	Melatonin	Oxygen
14	20	1	Left	Chronic	Verapamil	Triptan
						Oxygen
15	28	13	Left	Chronic	Verapamil	Oxygen
16	38	9	Left	Episodic	Verapamil	Triptan
					Prednisolone	Oxygen
					Tricyclic antidepressants	
17	47	16	Left	Episodic	Verapamil	Triptan
					Lithium	Oxygen
					Tricyclic antidepressants	
18^b^	36	7	Right	Chronic	–	–

All patients on preventive medication were on a stable treatment for at least 1 month prior to the first visit and throughout the duration of the study. Participants were instructed to abstain from all medication, apart from oxygen treatment, for at least 12 h prior to each scanning session. Duration CH = years passed since diagnosis of CH to beginning of the study; CH side = laterality of CH attacks; CH=Cluster Headache

^a^Participant excluded from behavioural analyses due to missing data

^b^Participant excluded from behavioural analyses and treatment response analyses due to missing data

**Table 2:** Behavioural data from Headache Diary (A), as well as from HADS and SF-36 questionnaires [29]

A. HEADACHE DIARY					B. PSYCHOMETRIC DATA					
Patients	Weekly CH attacks PRE GONB	Weekly CH attacks POST GONB	Improvement %	Group	Scale	Subscale	Mean ***N*** = 16	SD	T-test ***responders*** vs ***non responders***	
									t	sig
1	5	5	0	Non responder	**HADS**	Depression	9.06	5.26	0.416	0.684
2	35	16	54.2	Responder		Anxiety	8.88	4.455	0.326	0.749
3	10	0	100	Responder	**SF-36**	PF	82.5	19.235	−0.379	0.711
4	15	0	100	Responder		RP	15.63	23.936	−1.048	0.312
5	16	0	100	Responder		BP	27.13	24.816	−2.04	0.061
6	21	0	100	Responder		GH	76.38	50.599	−1.466	0.165
7	10	0	100	Responder		VT	37.88	18.301	−1.041	0.315
8	14	14	0	Non responder		SF	47.781	30.2264	−1.604	0.131
9	35	7	80	Responder		RE	37.44	41.968	−0.782	0.447
10	70	31	55.7	Responder		MH	57.5	23.905	−1.792	0.095
11	28	28	0	Non responder						
12	14	0	100	Responder						
13	4	4	0	Non responder						
14	38	36	5.2	Non responder						
15	4	6	−50	Non responder						
16	6	18	−200	Non responder						
17	35	31	11.4	Non responder						

Results from Headache Diary included 17 patients as one of the patients included in the MRI data analysis failed to provide results. Similarly, two patients failed to provide HADS and SF-36 responses, and therefore 16 patients were included in the independent samples t–test (i.e. eight responders and 8 non-responders). CH=Cluster Headache; GONB = Greater Occipital Nerve Block; HADS = Hospital Anxiety and Depression Scale; SF-36 = MOS 36-Item Short-Form Health Survey. Alpha = 0.05

### HADS and SF-36: responders vs non-responders at baseline

Due to missing data in questionnaires from two patients, data from a total of 16 participants, eight *responders* and eight *non-responders* were included in this analysis (for a summary of results, see Table 2B). Anxiety and depression measures reported by patients were on average between 8 and 10, an interval that corresponds to the category ‘borderline abnormal’ as specified by HADS score interpretation standards. Quality of life (QoL) scores represent the percentage of total possible scored achieved. We found the lowest scores (below percentile 40) in QoL subscales ‘role limitations due to physical health’, ‘energy/fatigue’, ‘role limitations due to emotional problems’ and ‘pain’. Independent samples t-test revealed no significant difference between *responders* (*n* = 8) and *non-responders* (n = 8) for all HADS and SF-36 subscales (all *p*’s > 0.05, Table 2B). Thus, independently of treatment outcome, *responders* and *non-responders* did not differ in depression and anxiety symptoms or quality of life measures at baseline.

### Imaging data

#### Regional CBF changes across patients: perfusion before vs after treatment

**Fig. 2:**
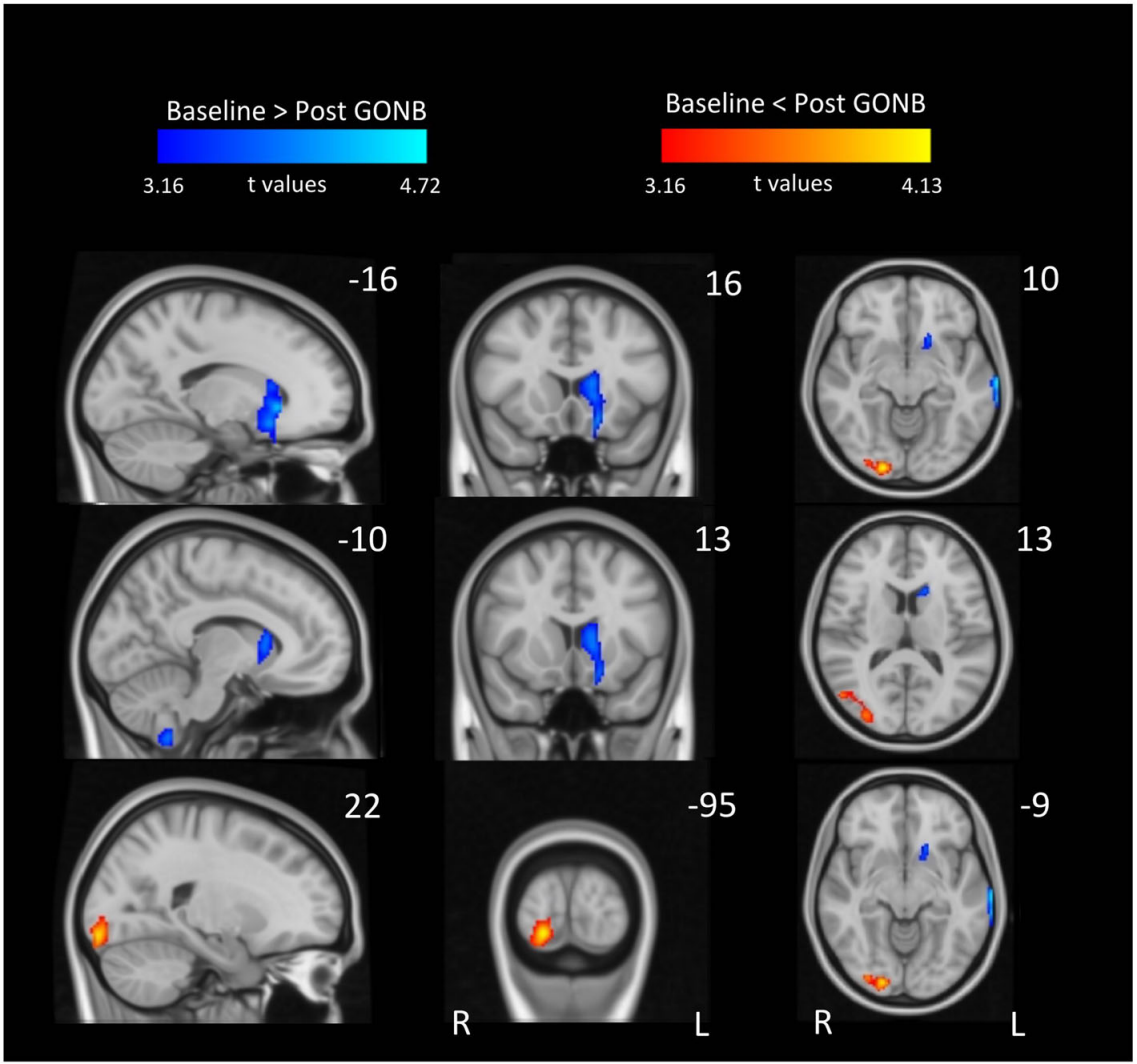
Local decreases (blue) and increases (red) of rCBF across all CH patients following GONB. Brain areas in blue include medial temporal gyrus, cerebellum and substructures of basal ganglia including caudate and putamen. Cluster in red colour corresponds to secondary visual cortex. Data included four CBF maps per patient and session. All clusters are significant at *p* < 0.05 (FWE corrected; initial height threshold set to 0.001). GONB = greater occipital nerve block; R = right; L = left

We sought to determine differences in rCBF between scans before and after GONB treatment for each patient. Repeated-measures ANOVA indicated a main effect of Treatment (i.e. pre vs post GONB); patients presented local decreases in rCBF after treatment across three main clusters in the left hemisphere, including posterior temporal gyrus, cerebellum and caudate, in comparison to the post GONB session. In contrast, increases in rCBF after treatment across patients were identified in the right secondary visual cortex (see Fig. 2 and Table 3). Exploratory results at a more liberal cluster-forming threshold (*p* < 0.005) revealed additional decreases in rCBF after treatment in thalamus bilaterally, right hypothalamus and ventral tegmental area (VTA), pons, substantia nigra bilaterally and cerebellum (Fig. 5).

**Table 3:** Summary of peak coordinates across all contrasts

Contrast		Cluster	Side	Peak coordinates (MNI)			Cluster size	t	***P***_**(FWE)**_	Cohen’s d	Hedge’s g
				x	y	z					
**Across all CH patients**	Pre > Post GONB treatment	medial temporal gyrus	left	−70	−20	−14	347	4.72	0.017	0.39	0.39
		cerebellum (lobule ix extending to lobule viii)	left	−6	−54	−60	351	4.48	0.016	0.37	0.37
		caudate extending to putamen	left	−16	22	−4	430	4.34	0.006	0.36	0.36
	Pre < Post GONB treatment	secondary visual cortex (ba18)	right	20	−92	−12	618	4.13	0.001	0.35	0.34
**Responders vs Non-responders to GONB at BASELINE**	Responders > Non-responders	lateral occipital cortex	right	38	−74	20	333	5.32	0.008	1.29	1.28
	Responders < Non-responders	posterior cingulate gyrus extending to primary motor cortex	right	2	−30	48	448	5.18	0.002	1.26	1.24
**Responders vs Non-responders to GONB AFTER treatment**	Responders > Non-responders	superior lateral occipital cortex	right	38	−74	20	1508	7.24	< 0.001	1.76	1.74
	Responders < Non-responders	posterior cingulate cortex	right	−4	−26	18	272	4.41	0.034	1.07	1.06
		middle temporal gyrus	left	−68	−46	2	312	5.03	0.019	1.22	1.21
	Responders>Non-responders (SVC)	medial prefrontal cortex	left	−12	52	14	115	5.56	0.015*	1.35	1.34
**CH patients vs Healthy controls**	CH Patients > Health controls	cerebellum (lobule viii)	left	−14	−62	−42	219	4.82	0.025	2.15	2.08
		hippocampus	left	−34	−30	−14	756	4.79	< 0.001	2.13	2.06
	CH Patients < Health controls	orbitofrontal cortex	right	22	56	−6	1896	7.97	< 0.001	3.55	3.43
		rostral anterior insula	right	52	18	2	218	6.77	0.025	3.02	2.92
		middle temporal gyrus	right	54	−18	−16	372	6.45	0.002	2.87	2.78

*CH* cluster headache, *GONB* greater occipital nerve block, *BA* Broadman area, *SVC* small volume correction

#### Predicting treatment response: regional CBF differences between responders and non-responders at baseline

**Fig. 3:**
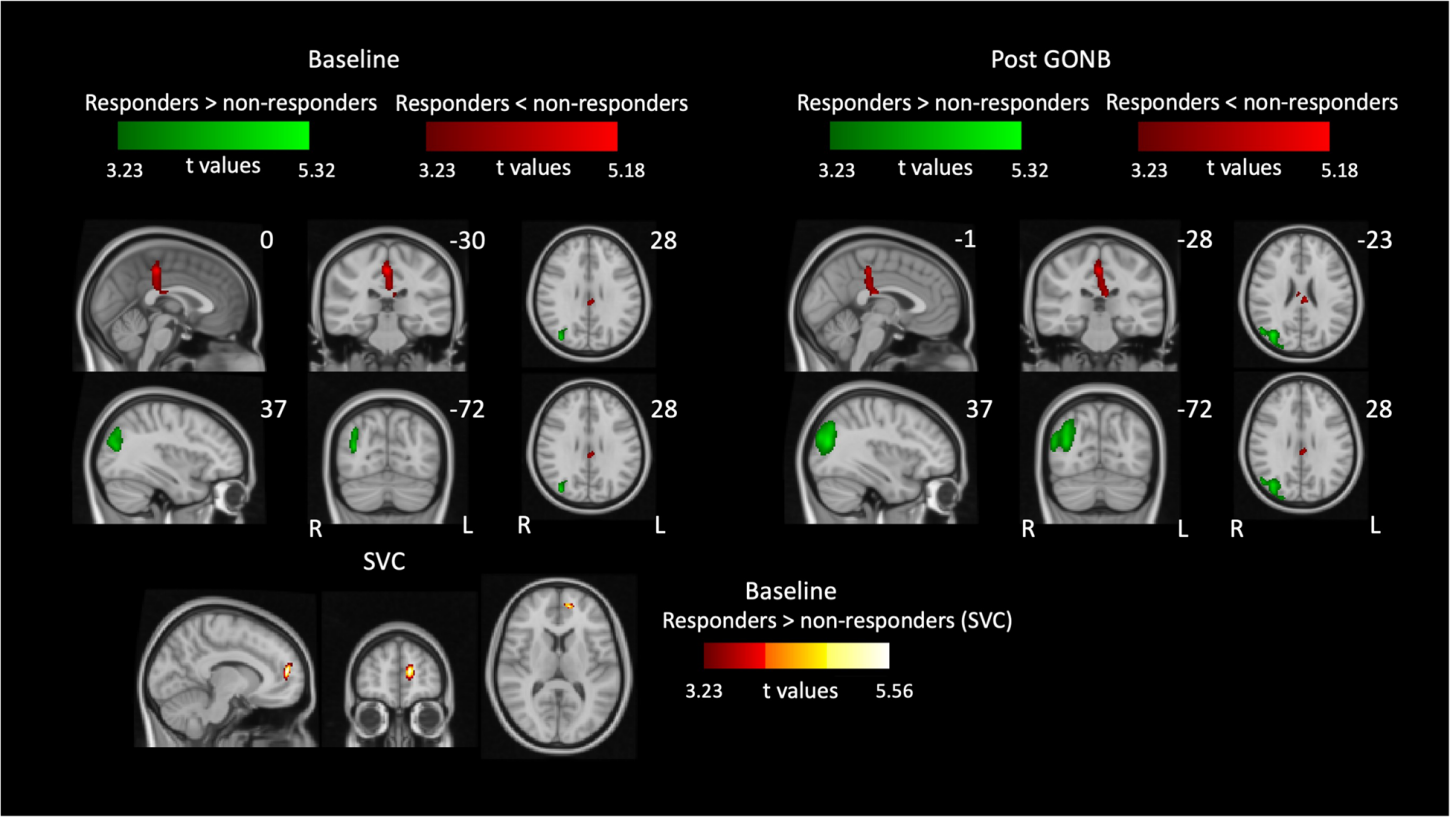
Differences in rCBF between the *responders* and the *non-responders* groups of CH patients at baseline (left panel) and following GONB (right panel). Increased local perfusion in *responders* (green) is observed in the LOC both prior and after GONB, being extended in the post GONB session. Increased local perfusion in *non-responders* comprised the PCC and the PMC in both sessions. Lower panel shows increased activation in mPFC for the *responders* group after performing small volume correction (SVC). Data included four CBF maps per patient and session. All clusters are significant at *p* < 0.05 (FWE corrected; initial height threshold set to 0.001). GONB = greater occipital nerve block; R = right; L = left

Contrasts comparing CBF maps at baseline (i.e. before GONB treatment) in *responders* versus *non-responders* group showed that patients who responded to treatment had greater rCBF in the right lateral occipital cortex, and lower rCBF in right posterior cingulate gyrus.

(Fig. 3, Table 3). Our hypothesis-led analysis (i.e. SVC) to test for prefrontal cortical CBF differences between responders and non-responders indicated that patients who responded to GONB treatment had greater rCBF at baseline in left medial PFC (mPFC), compared to patients who did not experience a substantial improvement after treatment (*p*FWE = 0.015, t-score = 5.56, 115 voxels).

#### Regional CBF differences between responders and non-responders after GONB treatment

With the purpose of acquiring a better understanding of differences between *responders* and *non-responders*, we compared CBF for both groups across the whole brain after treatment. Following GONB, patients who responded to GONB showed greater rCBF in right lateral occipital cortex, as well as lower rCBF in right posterior cingulate cortex and left middle temporal gyrus than patients who did not respond. SVC in the PFC did not show any significant differences between both groups following treatment.

#### Regional CBF differences between CH patients and healthy controls

**Fig. 4:**
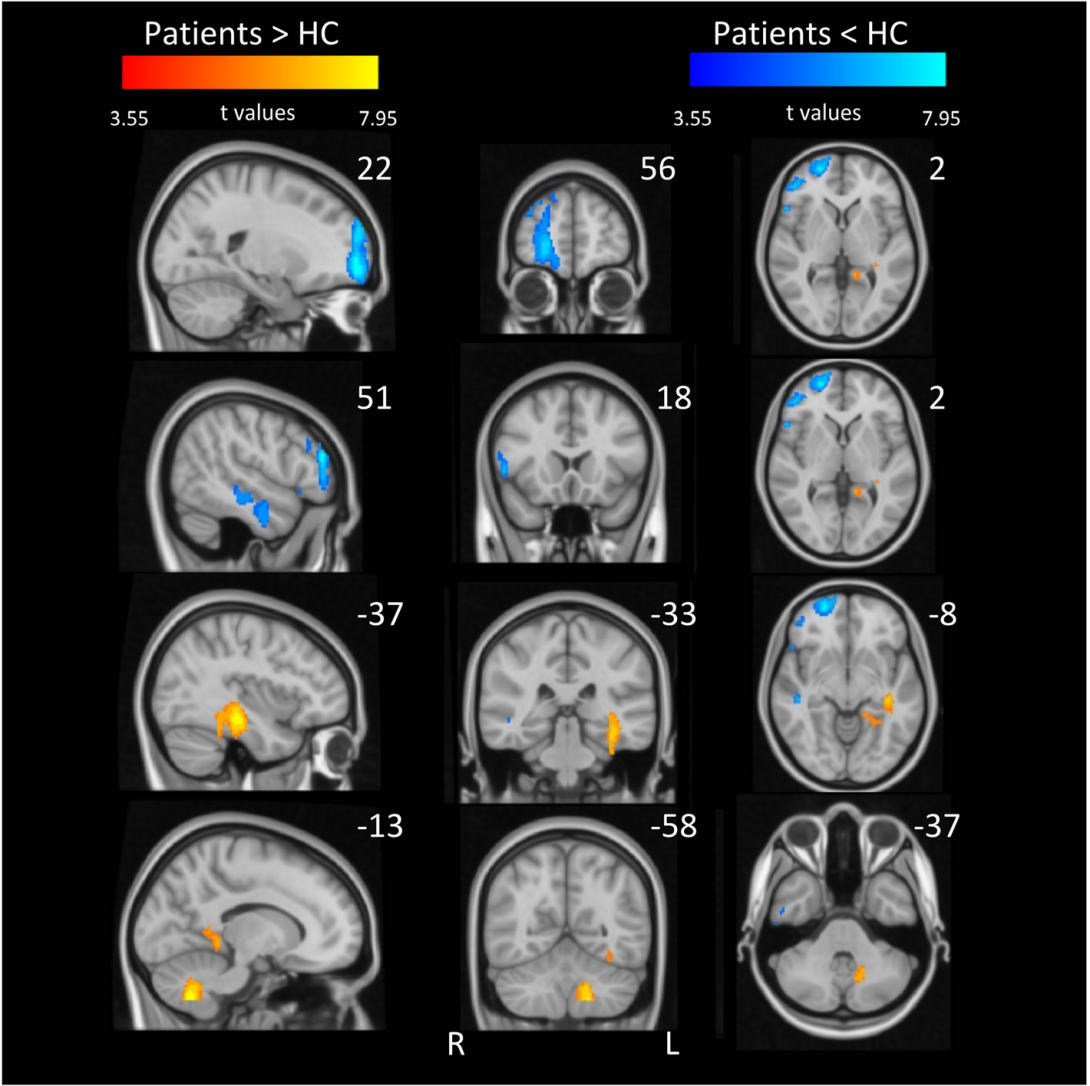
Local increases (red) and decreases (blue) in CBF across all CH prior to GONB in comparison to healthy controls. Brain areas in red include cerebellum and hippocampus. Clusters in blue colour corresponds to OFC, primary auditory cortex, insula and MTG. For these contrasts, data included one CBF map per patient and healthy individual. All clusters are significant at p < 0.05 (FWE corrected; initial height threshold set to 0.001)

We compared pre-treatment rCBF maps with those from a subgroup of healthy individuals (Fig. 4). Relative increases in rCBF in patients, compared to healthy control participants were observed in lobule VIII of left cerebellum and left hippocampus. Comparative reductions in the patient group were identified in the right orbitofrontal cortex, rostral anterior insula and middle temporal gyrus (Table 3).

## Discussion

We examined rCBF changes following GONB in CH patients, using pCASL fMRI imaging. We explored differences in rCBF between patients who responded to treatment and patients who did not respond, obtaining, to the best of our knowledge, the first evidence of rCBF differences that may act as predictors of GONB treatment efficacy in CH. Finally, we compared patients’ rCBF maps at baseline with matched healthy controls, in order to provide further meaningful information on the pathophysiology of CH. We discuss our findings across these three examinations and propose future directions of research acknowledging methodological caveats.

### The mechanisms of action of GONB

**Fig. 5:**
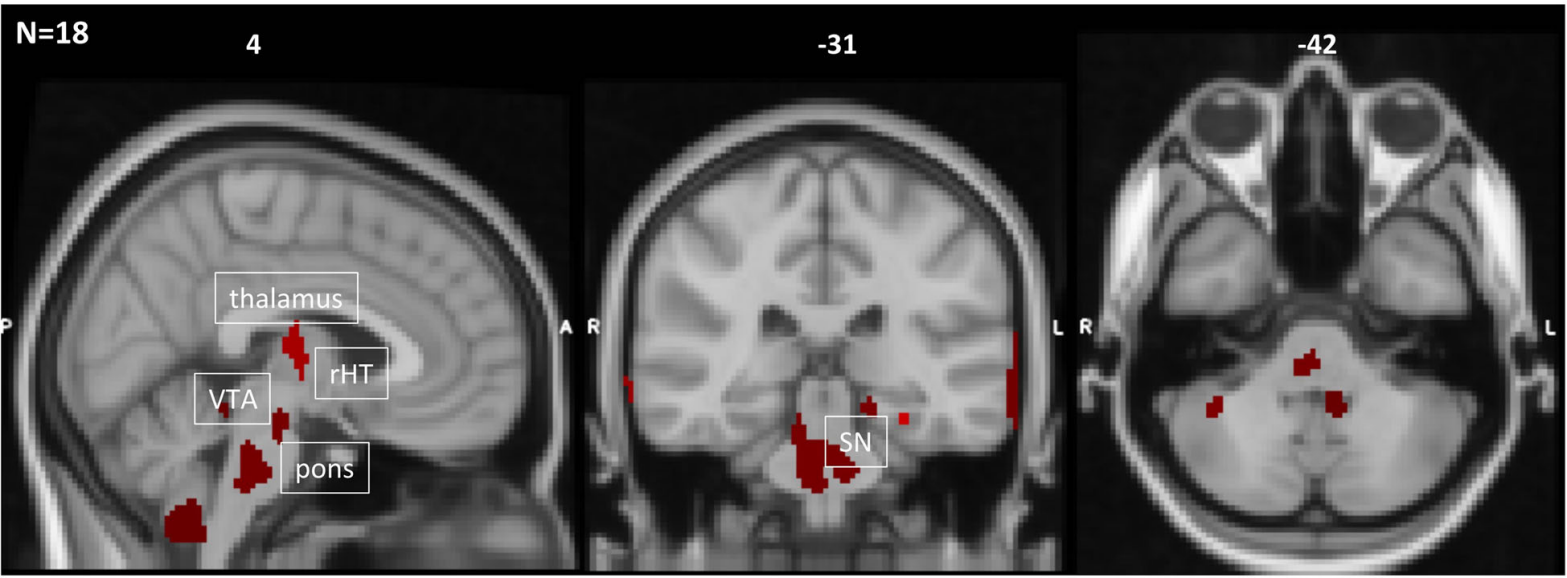
Exploratory results from local decreases in CBF across all CH patients following GONB at more relaxed initial cluster-forming height threshold. Data included four CBF maps per patient and session. All clusters are significant at *p* < 0.005 (uncorr). rHT = right hypothalamus; VTA = ventral tegmental area; SN = substantia nigra. Coordinates are represented in MNI space

We successfully identified rCBF changes in CH patients following GONB administration. Firstly, we observed rCBF reductions in lobule VIII of left cerebellum in all patients following treatment as well as in comparison to healthy controls. The connection between cerebellum and other structures commonly linked to CH mechanisms, such as VTA and hypothalamus, has been previously demonstrated in healthy individuals and CH patients and decreases in cerebellar metabolism in CH patients during hypothalamic deep brain stimulation have also been described [30]. Comparably, vagus nerve stimulation, currently used for suppressing CH attacks [31, 32], can provoke changes in rCBF in the inferior cerebellum in epileptic patients [33], and its mechanism of action has been linked to changes in the hypothalamic-pituitary-adrenal axis [34]. Taken together, our results support the theories of a modulatory function of the hypothalamic-cerebellar pathway in CH. Despite not observing perfusion changes in these midbrain areas in our data, such changes did emerge when using a more liberal cluster-forming threshold, commonly used in previous MRI literature (Fig. 5).

We observed increases in rCBF in right secondary visual cortex after GONB across patients. In contrast, perfusion decreases in primary visual cortex after GONB, as well as in comparison to healthy controls have been previously reported [35]; the authors speculated that these differences could be due to the existence of visual aura in CH patients. However, since we scanned patients during asymptomatic headache-free periods, interpreting our results in relation to aura is challenging. Nevertheless, *responders* to GONB demonstrated greater rCBF both at baseline and following GONB, compared to *non-responders,* in the right lateral occipital complex (LOC). The LOC has been shown to modulate pain memory [36] and it has been suggested to be involved in the lateralisation of CH attacks [37]. In fact, evoked trigeminal pain in healthy individuals interrupts visual encoding in this group of areas [38]. Our results, in line with these findings, indicate that the pathophysiology of CH may well extend beyond areas commonly associated with pain experience [39], pointing towards the integration of pain structures and superior areas of the visual system in the pathogenesis of CH.

Finally, following GONB we observed decreased perfusion across patients in the dorsal striatum. The striatum is well known for playing a major role in endogenous analgesia [40]. It is directly connected to the trigeminal nucleus caudalis, which is the primary target for GONB afferent inhibition [41] and its analgesic effect relies on activation and propagation of dopamine D_2_ receptors towards the trigeminal nerve via basal ganglia. Specifically in CH, increased axial diffusivity in the caudate nucleus in CH patients compared to controls has been reported [42] suggesting altered neural pain-related plasticity in these patients. Neuropeptide studies may shed further light into how these results relate to a CBF reduction in the striatum after GONB, as they may provide with a comprehensive description of the molecular processes taking place between the striatum and the trigeminovascular nociceptive pathways [43]. Admittedly, our results from perfusion before vs after GONB comparisons yield a modest effect size, as indicated by Hedge’s g figures that gravitate between 0.35 and 0.39 (Table 3), which may well be a direct consequence of our relatively low sample size; however, this first attempt to characterise mechanisms of GONB responses in an arguably rare clinical cohort like CH patients should facilitate future highly powered hypothesis-driven versions, including replications, of the present experimental design. It is important to stress that the CBF changes following GONB discussed above are unlikely to relate to the effects of corticosteroid intervention, not only because medication remained stable throughout the study and therefore any variability would be across patients and not within-participants, but also because only two patients included in the analyses were on prednisolone treatment, precluding a main effect of the drug to emerge at group level. One could also argue that the reduction of CH attacks following treatment across ECH patients could be due to the natural remission of their bout; this was accounted for in out design, and accordingly, we made sure both study visits were scheduled before their bout was expected to end, aiming always for the first half of their bouts. Nevertheless, and despite our best efforts, we cannot rule out that in some exceptional cases the bouts ended earlier than usual, a factor that always should be taken into account when determining responses to treatment in episodic CH patients.

### Prediction of treatment response

It remains critical to identify brain characteristics at baseline that may indicate the likely efficacy of GONB in suppressing CH attacks in individual patients. We investigated this question via examination of differences in rCBF at prior to GONB between *responders* and *non-responders*.

In line with our a priori hypothesis, we observed greater CBF in the medial PFC in *responders* at baseline compared to *non-responders.* This result was not driven by differences in depression, anxiety or quality of life measures between the two groups. The role of the medial PFC in anticipation of placebo analgesia has already been described in healthy volunteers,^46^ and the extent of central hyperalgesia is negatively correlated with activity in this area [44]. Additionally, placebo effects have been linked to differential treatment responses in CH [45], however, imaging evidence on placebo effects in CH has yet to be reported. A limitation of our study is the absence of placebo control arm, but it is arguable that the medial PFC is indeed involved in mediating response in the active treatment arm. The implication that the medial PFC may be involved in mediating response to both active and placebo treatment needs further investigation.

We identified reduced local CBF in the posterior cingulate cortex (PCC) that extended to the primary motor cortex in *responders* in comparison to *non-responders,* that were unaltered by GONB. The PCC is involved in integration of memories, motivational-affective components [46], and ruminating thoughts during pain [21]. Moreover, enhanced perfusion compared to healthy individuals in this area has been suggested to be an increased orientation of attention towards pain in osteoarthritis patients [47]. Together, our results suggest that differences in the PCC might relate to patients’ psychological states in relation to CH. We speculate that more negative beliefs and ruminating thoughts towards the condition and the future are likely to relate treatment efficacy.

We identified decreases in rCBF in left medial temporal gyrus (MTG) across patients following treatment, as well as lower rCBF in the *responders* group compared to *non-responders* at baseline. Increased perfusion in MTG in CH patients when comparing scans in bout vs out of bout has been reported [13]; further, reduced FC between hypothalamus and MTG [48] and reduced GM in MTG [29] have been shown in CH patients compared to healthy individuals. Importantly, MTG activation seems to be involved in inhibitory function during conditioned pain modulation [49] and it has been linked to pain recognition in others [50]. Since the MTG is classically linked to recognition and retrieval if concepts, our results, together with existing evidence, hint that impaired meaning attribution of pain-related information in the MTG might play a role in the pathophysiology of CH.

### CH patients vs healthy controls

We observed greater rCBF in the orbitofrontal cortex (OFC) in healthy controls in comparison to patients. It has been suggested that reduced GMV in OFC contributes to poorer top-down inhibitory control of pain signals in chronic pain, including CH [14]. These findings suggest that perturbations in OFC may impair chronic pain patients’ capacity to manage afferent nociceptive signals.

We also identified greater rCBF in the dorsal hippocampus in CH patients compared to healthy controls. GMV reductions in CH patients compared to healthy controls have been reported [16], that develop and change with time and disease stage, suggesting that the hippocampus could be involved in pain memory, and its activation is related to pain expectancy and harm avoidance [51]. The hippocampus is one of the main mediators of anxiety in pain processing [52]. It is plausible that anxiety-related personality traits, as indexed by our borderline HADS results, are playing a role in the emergence CH symptoms by priming memories of previous headache attacks and facilitating pain states. Furthermore, we observed decreased rCBF in the rostral anterior insula in the CH patients group compared to healthy controls at baseline. Decreases in GMV in the anterior insula in CH patients out of bout versus healthy controls have been previously reported [16]. Some authors argue that the rostral anterior insula is a specific locus for clinical pain, regardless of the pain condition [53], suggesting a distinct pathway impairment in these patients. Qiu et al. [54] found decreased FC between the hypothalamus and the salience network, of which the anterior insula is a key component, in pain-free CH patients in bout compared to healthy controls. Importantly, the hypothalamus is heavily involved in stress regulation through the hypothalamus-pituitary-adrenal axis [55]. Our results stand in line with these claims, suggesting that a combination of higher stress response facilitation towards pain, alongside with impaired stress-related affective response control may be elements that ultimately, contribute to the chronification of headache attacks.

Despite the fact that our results involving differences between *responders* and *non-responders*, as well as between CH patients and healthy controls are indeed derived from a low number of participants, the corrected effect size estimations for each of the contrasts are largely above the accepted cut off for an effect considered as ‘large’ (e.g. Hedge’s g > 0.80), serving as a first indicator of the robustness of the data discussed above. Nonetheless, forthcoming studies including a larger number of patients and healthy controls may replicate these findings and shed further light into perfusion patters that may reliably act as predictors of GONB response in CH patients.

## Conclusions

In summary, our results indicate that the pathophysiology of CH includes, but is not limited to, brain areas typically linked to pain perception; while changes in brain perfusion after GONB point out as possible main targets areas innervated by the trigeminal nerve (i.e. cerebellum, striatum, visual cortex), we propose that there is a heavy psychological component that might be driving treatment responses through poor anxiety and stress response regulation, attentional bias towards pain, and ruminating thoughts; our results point to differences in areas previously associated with these psychological states at baseline. Future research may elucidate whether response to GONB may be improved by combining it with therapies focused on controlling negative thoughts towards pain promoting cognitive flexibility. Likewise, further investigation of GONB responses including placebo-controlled designs might disentangle differential responses to treatment. Overall, our findings provide further characterisation of underlying brain mechanisms in CH that extend beyond the traditional midbrain hubs widely discussed in the literature.

## Data Availability

All data were acquired at the Centre for Neuroimaging Sciences at King’s College London. Composite groupwise statistical maps derived study analyses are available from the corresponding author SM on reasonable request.

## Abbreviations

CH: Cluster Headache
GONB: Greater Occipital Nerve Block
rCBF: Regional Cerebral Blood Flow
MRI: Magnetic Resonance Imaging
BOLD: Blood Oxygenation Level Dependant
ASL: Arterial Spin Labelling
pCASL: Pseudo Continuous Arterial Spin Labelling
GMV: Grey Matter Volume
PFC: Prefrontal Cortex
HADS: Hospital Anxiety and Depression Scale
SF-36: 36-item Short Form Survey
FSE: Fast Spin Echo
FSL: FMRIB Software Library
MNI: Montreal Neurological Institute
SPM: Statistical Parametric Mapping
FWE: Family Wise Error
ANOVA: Analysis of Variance
CSF: Cerebrospinal Fluid
SVC: Small Volume Correction
ROI: Region of Interest
VTA: Ventral Tegmental Area
LOC: Lateral Occipital Cortex
PCC: Posterior Cingulate Cortex
MTG: Medial Temporal Gyrus
OFC: Orbitofrontal Cortex
